# The Sectional Teachings of Medicine

**Published:** 1849-07

**Authors:** Daniel Stahl

**Affiliations:** Quincy, Illinois


### ARTICLE V.

*The Sectional Teachings of Medicine.*—By DAN'L STAHL, D.M.

QUINCY, ILLINOIS, APRIL 11, 1849.

*Prof. W. B. Herrick:*

*Dear Sir:—*

Is "sectional teaching" of the practice of medicine useful or necessary? or "to treat diseases understandingly, is it requisite for the student to be educated in the localities where they prevail?" These are questions which are now asked and discussed in the circles of Medical men, and in the medical papers of the United States, and concerning which think we ought to come to some understanding. Having no connection, directly or indirectly, with any medical institution, and having no private ends to accomplish which, in the remotest manner, could possibly bias my judgment, I trust that you will consider the opinions which I shall take the liberty to advance here, as the expressions of an honest con viction—a conviction which has been forced upon me in the trying school of an experience to which I look back with any other than pleasurable feelings.

The physical or material man is everywhere the same; his organization, with but very slight qualifications, is the same on the sandy plains of Arabia, and on the summit of the Rocky Mountains; on the banks of the Rhine and on those of the Colorado;—everywhere man presents the same number of organs, in (mutatis mutandis) the same shape, and organic chemistry finds the same elements in their composition. The functions of this complex organism are also everywhere the same in the whole species of that wonderfully constructed animal *homo*. When, therefore, we wish to examine and to study the construction, the composition, and the functions of this living machine—when we wish to study *anthropology*—it matters little *where* we are taught, if only taught *well*—whether at Paris or Vienna, at Riga or Philadelphia. But it is quite different with the study of pathology and therapeutics. Pathological change and remedial agents differ, and must necessarily differ, according to climate, and even, sometimes, according to locality. Does it follow as a matter of course, that, because man's physical organization is everywhere the same, he is, therefore, subject to the same diseases, no matter whether he inspires the mephitic air of the jungles of Bengal, or the balmy atmosphere of the valleys of the Rhine? By no means. The experience of those physicians who have visited different climates will testify to the contrary; works written by medical men in different countries will testify to the contrary. Although the principle of self-preservation is in man, and, consequently, that of reaction against inimical influences, yet the *constant* actions of these external impressions will and do produce an effect either on the whole system or on single organs primarily, and, by sympathy or otherwise, on the whole system. Thus we find that the inhabitants of warm climates are more subject to hepatic affections; in some parts of the world there is goitre, in another plica polonica endemica; in some countries, we find yellow fever and never see a case of the plague; in others we find the plague in its most horrible form and never have an opportunity of seeing yellow fever. Without extending these illustrations any further, I think I have proved sufficiently that our climate and even our locality can predispose to and produce ills from which the inhabitants of another climate or locality are perfectly exempt, and that hence "sectional teaching," in the liberal sense of the word, is indispensable, unless the practitioner prefers the slow process of experimenting on his patients till he has supplied the advantages of "sectional teaching," by his own observations at the bedside and at the dissecting table. To render the illustration, or, if you will, the argument, still more striking, let us suppose a young man of liberal education and all the other qualifications necessary to make him a good physician, from Mississippi or Louisiana, to have studied medicine at Paris or Berlin, and returning to his native state with the most honorable credentials of his proficiency, learning, and skill in his profession, think you that he will be able to treat yellow fever, congestive fever, and all the sequalæ of these endemic fevers with the same ready tact, skill, and familiarity as he who, during his pupillage, watched at the bedside of patients with such maladies, and who has been taught by men who have observed and treated thousands of such cases? You certainly cannot think so, because you must know that the former had not only no opportunity to see such cases at Paris or Berlin, but they were not even described to him by men who are supposed to have seen them, but by those who have probably *read* outlines of these pernicious fevers, and from these outlines formed a picture of them in their minds—in short this unfortunate Mississippian or Louisianian with his great medical acquirements cannot diagnosticate and treat these endemical diseases of his native state with as much skill and efficiency as he who availed himself of "sectional teaching," because he was taught by men who cannot communicate practical instruction either at the bedside or from actual knowledge. Book-learning alone will not do either with the teacher or practitioner; both must have "seen and handled."

You perceive then, that although "all men are created equal," "sectional" influences produce "sectional" diseases, the nature, course, and treatment of which can, from the peculiarity of these influences and their effects on the organism, be taught practically and to useful ends only by those who can speak from *experience*. An ounce of experience, with a little learning, both of the teacher and pupil, is worth more to the patient than never so many pounds of learning without experience. Ask any one of the professors of the theory and practice of medicine in the western medical colleges what, aside from his general medical education, renders him competent to instruct his pupils in the symptomatology, course and treatment of the diseases peculiar to the western country as modified by the influence of its climate, and if he does not answer "*practical* knowledge," he is incompetent to the task he has assumed, and he violates the trust reposed in him. The fact is, the teacher must have the true picture of the disease he treats of vividly in his mind, and so he must have the picture of all the phases this disease may assume, and such a picture can be impressed on his mind by observations, by the perception of his senses, and by no other means; and all the sophistical talk about the general principles of medicine, etc., etc., in order to get around the necessity of this "sectional teaching," I consider as coming from men who do not know better or who will not know better. What renders the works of Eberle, Bell, and other American authors, and the monographs (as far as the practical part is concerned) of Dr. S. Cartwright so useful and valuable to us but the truly practical instructions they contain concerning the diseases of this country? and could these have written as they have if they had not seen and treated these diseases? Certainly not. Dr. Bell, in his "lectures," frequently appeals to *his experience* in deciding points of controversy in relation to the treatment of our diseases, and in the preface to his "Bell and Stokes' Lectures" he says—and this I cite as a potent argument for "sectional teaching,"—"and by steadily bearing in mind the wants and expectations of the American practitioner for information respecting the fevers of the United States and analagous climates, rather than those of European hospitals, camps, and jails, less disappointment, it is hoped, will be felt at my abbreviations on this head, (the literature and history of fevers). I have curtailed to some extent my former lectures on congestive fever, but have still retained those distinctive features which imparted to them that interest in the minds of the physicians of the South and West, which I was sanguine enough to anticipate when I first took up the subject," etc., "*for a complete elucidation of the nature and treatment of which* (Southern and Western fevers) *they* (the physicians of the South and West,) *must not look to the hospital statistics nor to the collegiate teachings of Europe*," etc. And in an article in the October number, 1846, of "The Bulletin of Medical Science," (vol. iv, No. 10,) this same distinguished writer, (Dr. John Bell,) says, pp. 344 and 345, in reply to Dr. Cartwright's remark, that Dr. Bell has written about as well on congestive fever as any other northern writer who has gone to the north of Europe, where these types of fever never prevailed, to get information, instead of coming South or turning to Hippocrates—"It is not easy to reply to an assertion which could not have been made by any person in his right senses, who had ever read the lectures on congestive fever. He would then have learned readily enough that Dr. Bell did *not* go to the north of Europe to get information on this subject, but that he *did* go to the south, not only in the sense intended by Dr. Cartwright, viz., by consulting those who had southern experience, but, still more, that he went, himself, south, and that by many degrees nearer the equator than either Dr. Cartwright or any of his state rights medicine party. Dr. Bell studied medicine and observed disease in Virginia," etc.

Many of the diseases of the Western States, particularly of the valley of the Mississippi, require a different treatment from similar diseases of Europe and even of the Atlantic States; others, again, we have here, which are absolutely unknown either in Europe or in New England. If the doctrine of the catholicity of medicine be correct it ought to be indifferent whether a physician is educated at Berlin, Paris, or Chicago, provided he obtains good and sufficient instruction, and this instruction received in any one of these places, ought, consequently, to enable him to practice his profession any where on the habitable globe. But is this attainable? I think not; and to prove this, let us suppose a physician fresh from the best university and hospital of Europe to visit a patient laboring under that dreadful scourge of American infants, *cholera infantum*, and what will be his diagnosis? Anything but the real disease. Or let him be called to a case of *milk-sickness*, (N. B. Do our eastern brethren know anything about this?) and is it at all probable that he can diagnosticate and treat the case correctly? It is almost impossible, because even systematic works on the theory and practice of medicine rarely contain any notice of this disease—a disease which the practitioner in Kentucky, Indiana, and other western states has so often to encounter. Admitting that these two complaints are American and that the European cannot know them except by reading, and we can therefore not pre-suppose him to possess any knowledge of them, let us invite him to the bed-side of a patient, who labours under a malady that is found both in Europe and America. I mean the disease which is here vulgarly called *winter fever*. I doubt not but what our stranger will properly diagnosticate the case; but finding the patient the resident of a log cabin standing upon the banks of one of our western rivers, (and there or in similar locations is where we mostly find these cases of pneumonia notha,) does any western physician suppose that he can treat it with safety? I, for my part, doubt it; at least I would not trust myself in his hands until he had gained the knowledge of its course, etc., by experience.

I could extend these illustrations, but I trust that these few will be sufficient to prove the necessity of acquiring a knowledge of those diseases, which it will be the future lot of the practitioner to treat, not only from men who from *experience know them*, but also from observation at the bed-side.

Let one of those who maintain that "the great principles of pathology and therapeutics can never be sectional," (and this position I will say, en passant, I don't altogether dispute, I only dispute the "universal application" of subordinate principles:) be taken sick of yellow or congestive fever, let him be surrounded by Schoenbien, Louis, Williams, Mitchel and Cartwright, and let him choose one of these to attend him professionally. Is it likely that he would take the Berlin or Paris, or even the Philadelphia Professor, world renowned as each of them may be, when he has at his command the services of a Cartwright who has spent the best years of his life in observing, studying and treating these diseases? I trow he would trust to "sectional medicine," and sectional treatment too. Or if this same man—this opponent of "sectional teaching"—wishes his son to study medicine with a view of locating at Natchez, New Orleans, or St. Louis, or even at Quincy, would he not advise him to obtain instruction in relation to the diseases incident to these localities from physicians who know them from *experience*, in preference to those who know them but by *reading*, or judge of them by "analogy." Most assuredly he would.

To avoid misunderstanding, I must beg you to consider my advocacy of "sectional teaching" in that sense in which alone it can be reasonably defended, or is necessary or salutary. To say that the European schools, or the schools on the other side of the Alleghanies, cannot impart to our western physicians a good general scientific and practical medical education would be an absurdity; but, notwithstanding this concession, I think I have shown sufficiently in the preceding remarks that these European and Eastern Schools cannot teach their pupils the symptomatology, ætiology, course and treatment of our western diseases. As well as western physicians, western schools, and western hospitals; and I wish you to bear it in mind *that to this class of diseases* (to treat which, by the by, is two-thirds of the employment of a western phpsician) *I confine my advocacy of "sectional teaching*."

Accept, my dear Doctor, the renewed assurances of my respect and best wishes. DANIEL STAHL, M. D.

